# Zebrafish model of *KRAS*-initiated pancreatic endocrine tumor

**DOI:** 10.1080/19768354.2019.1610058

**Published:** 2019-05-10

**Authors:** Sekyung Oh, Joon Tae Park

**Affiliations:** a Department of Medicine, Catholic Kwandong University College of Medicine, Gangneung, Republic of Korea; b Institute for Biomedical Research, Catholic Kwandong University International St. Mary’s Hospital, Catholic Kwandong University, Incheon, Republic of Korea; c Division of Life Sciences, Incheon National University, Incheon, Republic of Korea

**Keywords:** Pancreatic endocrine tumor, zebrafish, *KRAS*

## Abstract

Pancreatic cancer constitutes a genetic disease in which somatic mutations in the *KRAS* proto-oncogene are detected in 95% of cases. Activation of the KRAS proto-oncogene represents an initiating event in pancreatic tumorigenesis. Here, we established a zebrafish pancreatic neoplasia model that recapitulates human pancreatic tumors. Toward this end, we generated a stable CRE/Lox-based zebrafish model system to express oncogenic *KRAS^G12D^* in the *elastase3I* domain of the zebrafish pancreas. Lineage tracing experiments showed that early *KRAS^G12D^*-responsive pancreatic progenitors contribute to endocrine in addition to exocrine cells. In this system, 10% and 40% of zebrafish developed pancreatic tumors by 6 and 12 months, respectively. The histological profiles of these experimental tumors bore a striking resemblance to those of pancreatic endocrine tumors. Immunohistochemical analysis including the endocrine cell-specific marker confirmed the pancreatic tumor region as a characteristic endocrine tumor. Taken together, our zebrafish model data revealed that pancreatic endocrine tumors originate from early *KRAS^G12D^*-responsive pancreatic progenitor cells. These findings demonstrated that this zebrafish model may be suitable as an experimental and preclinical system to evaluate different strategies for targeting pancreatic endocrine tumors and ultimately improve the outcome for patients with pancreatic endocrine tumors.

## Introduction

Pancreatic cancer comprises the fourth leading cause of cancer death among men and women in the United States, with most cases resulting in patient death (Jemal et al. 2008). Activation of the KRAS proto-oncogene represents an initiating event in pancreatic tumorigenesis, with 95% of pancreatic cancers in humans harboring an oncogenic *KRAS* mutation (Miyamoto et al. 2003; Park et al. 2008). Thus, because an activating mutation of the *KRAS* allele represents the most frequent genetic alteration associated with pancreatic cancer, the majority of genetically engineered mouse models are based on the *KRAS* mutation. Several mouse models for *KRAS*-mediated pancreatic cancer have successfully replicated the early and advanced forms of this disease (Aguirre et al. 2003; Hingorani et al. 2003; Hruban et al. 2006). However, successful translation of preclinical studies in mouse models toward the effective treatment of human disease has been inefficient (Kapischke and Pries 2008). To address this fundamental problem, it is necessary to develop a new platform that can significantly accelerate preclinical drug development.

The zebrafish has emerged as an excellent model organism in the study of cancer biology over the past several decades. General cancer characteristics such as genomic instability, invasiveness, and metastasis have been shown in zebrafish as well as mammalian tumors (Le et al. 2007). The optical transparency of the zebrafish embryo makes it possible to track and monitor transgenic tumors with regard to their initiation, progression, and metastasis (Beis and Stainier 2006; Liu et al. 2008). To maximize the likelihood of recapitulating human pancreatic cancer development in zebrafish, we have adapted reagents for CRE/Lox-based gene activation. However, no reliable promoters were available to ubiquitiously drive transgene expression in zebrafish. Notably, the zebrafish Ubiquitin b (*ubb*) promoter was recently established and shown to induce transgene expression in the vast majority of cell types and through all stages of zebrafish development, starting at the mid-blastula transition (Mosimann et al. 2011).

In this study, we used the CRE/Lox system to express an oncogenic *KRAS* transgene under the control of the zebrafish *ubb* promoter. After expressing the transgene in the *elastase3I* domain of the zebrafish pancreas, we interrogated the effect of *KRAS^G12D^* expression on the development of pancreatic cancer. Here, we first demonstrated that pancreatic endocrine tumors originate from early *KRAS^G12D^*-responsive pancreatic progenitor cells.

## Materials and methods

### Generation of transgenic zebrafish

All animal studies were reviewed and approved by the International Animal Care and Use Committee of Johns Hopkins Medicine (protocol number: 20120112001). Fish were raised and maintained under standard laboratory conditions. The following strains were established and/or utilized: *Tg (elastase3I:CRE;cryaa:Venus)* (herein ela3I:CRE) (Hesselson et al. 2011) and *Tg (ubb:Lox-Nuc-mCherry-stop-Lox-GFP::KRAS^G12D^*) (herein *LSL-KRAS^G12D^*). Larvae were anesthetized in 0.16% tricaine (A-5040; Sigma, St. Louis, Missouri, USA). Adult zebrafish were euthanized by induction of tricaine anesthesia followed by placement in an ice bath, consistent with recommendations of the Panel on Euthanasia of the American Veterinary Association.

### Analysis of tumor incidence in adult fish

Transgenic adult male *Tg* (ela3I:CRE) fish were outcrossed to a transgenic adult female *Tg (LSL-KRAS^G12D^)* fish. Embryos expressing GFP in the pancreas at 5 days post-fertilization (dpf) were raised. A random subset of fish was anesthetized and sacrificed at 3-, 6-, and 12-month time points for histologic evaluation.

### Immunofluorescence, immunohistochemistry, and trichrome staining

Immunofluorescence and immunohistochemistry analyses were performed on 5 μm paraffin-embedded sections as described previously (Lin et al. 2004). Primary antibodies used for immunofluorescence labeling comprised rabbit anti-carboxypeptidase A (CPA) (200-4152; Rockland Inc., Pottstown, PA, USA; 1:500 dilution), guinea pig anti-insulin (A0564; Dako, Carpinteria, CA, USA; 1:400 dilution), mouse anti-GFP (MAB3580; Millipore, Burlington, MA, USA; 1:500 dilution), and rabbit anti-PCNA (sc-7907; Santa Cruz Biotechnology, Dallas, TX, USA; 1:500 dilution). Secondary antibodies were Cy5-conjugated anti-rabbit antibodies (711-175-152; Jackson Immunoresearch Laboratories, West Grove, PA USA; 1:400 dilution), Cy3-conjugated anti-mouse antibodies (715-165-150; Jackson Immunoresearch Laboratories; 1:400 dilution), and Cy5-conjugated anti-guinea pig antibodies (706-545-148; Jackson Immunoresearch Laboratories; 1:400 dilution). Primary antibodies used for immunohistochemistry were rabbit anti-chromogranin A (ab15160; Abcam, Cambridge, MA, USA; 1:400 dilution), rabbit anti-GFP (A11122; Invitrogen, Waltham, MA, USA; 1:400 dilution), rabbit anti-phospho AKT (4060S; Cell Signaling Technology, Danvers, MA, USA; 1:400 dilution), and rabbit anti-phospho ERK (4370S; Cell Signaling Technology; 1:400 dilution 1:400). The secondary antibody was biotin-conjugated anti-rabbit (711-066-152; Jackson Immunoresearch Laboratories; 1:500 dilution). For ABC reaction, the ABC kit Vectastain PK-6100 from Vector Laboratories (Burlingame, CA, USA) was used. Masson-Trichrome staining was performed according to the manufacturer’s instructions (25088-100; Polysciences Inc., Warrington, PA, USA).

### Microdissection and quantitative polymerase chain reaction (PCR)

RNA was isolated from paraffin-embedded tissues on microscope slides using the Pinpoint Slide RNA Isolation System kit (R1007; Zymo Research, Irvine, CA, USA). RNA was reverse transcribed using the QuantiTect Reverse Transcription Kit (205310; Qiagen, Hilden, Germany). Expression of specific mRNAs was measured by quantitative real-time PCR using a SYBR Green-based method. Average fold changes were calculated by differences in threshold cycles (Ct) between pairs of samples. The primer sequences were as follows: insulin (F; GCTCTGTTGGTCCTGTTGGT, R; GGGCAGATTTAGGAGGAAGG), chromogranin A (F; GAAAACGATCCGGCTCATAA, R; TGGGCTCATCTCCACTCTCT), amylase 2a (F; GGAAACATTGAGAACTACCAG, R; GCCATAAACAGCAGACAGA), krt18 (F; AGTGGTAGCACAGGCGAGAT, R; AAGATCTTCTTGCGCAGCTC), and β-actin (F; CGAGCAGGAGATGGGAACC, R; CAACGGAAACGCTCATTGC).

### Statistical analyses

Statistical analyses were performed using a standard statistical software package (SigmaPlot 12.5; Systat Software, San Jose, CA, USA). Student’s *t*-test was used to determine whether differences were significant (**P < .01 and *P < .05).

## Results

### Generation of *Tg* (ela3I-CRE; LSL-KRAS^G12D^) fish

**Figure 1.**
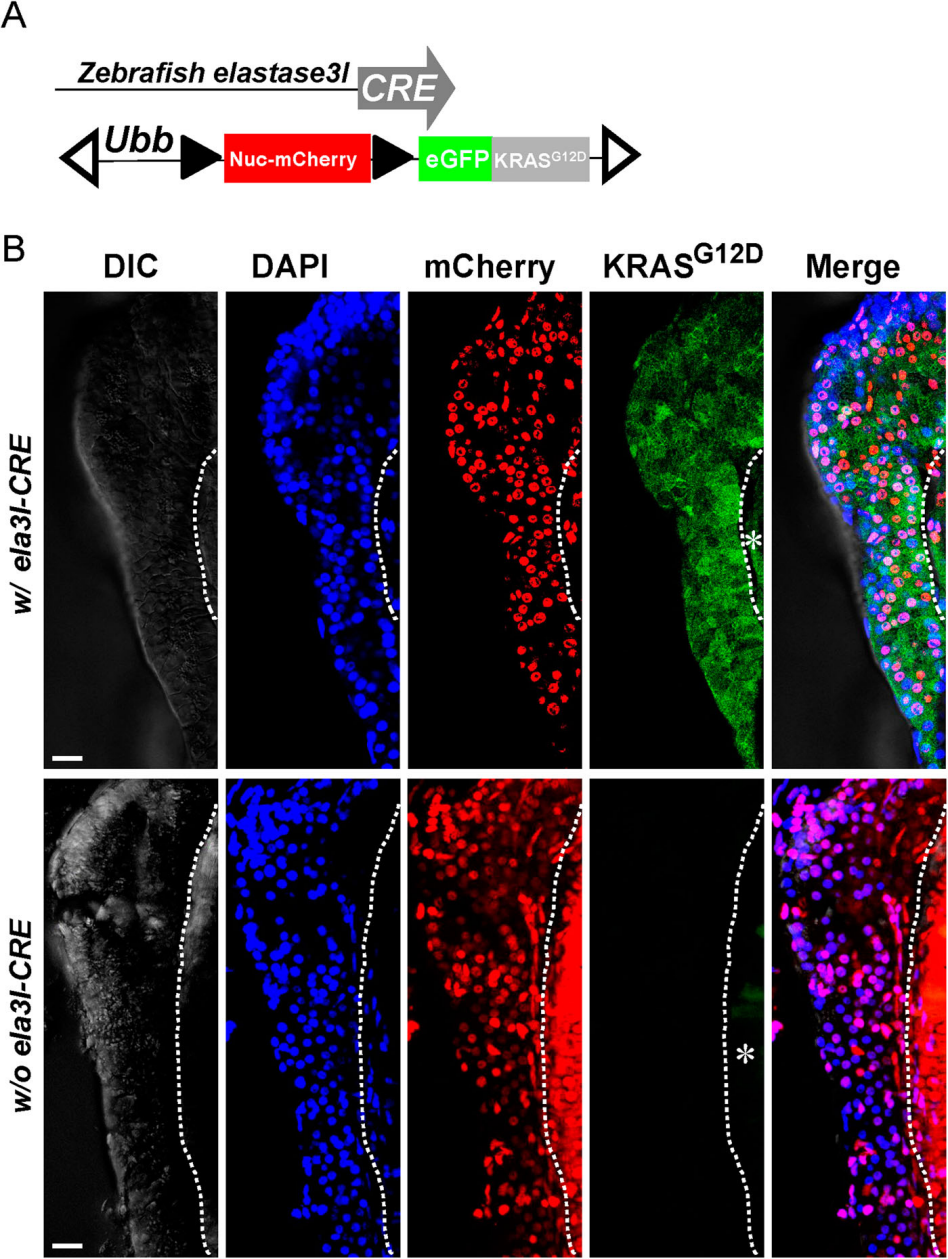
Generation of *Tg* (*ela3I-CRE; LSL-KRAS^G12D^*) fish. (A) Schematic diagram of *Tg (ela3I-CRE)* driver lines and *Tg (LSL-KRAS^G12D^)* reporter lines. Open triangles indicate Tol2 arms. (B) Confocal images of the microdissected pancreas (5 dpf) in *Tg* (*ela3I-CRE; LSL-KRAS^G12D^*). GFP signal was detected mainly in the membrane of the pancreas and partly in the cytoplasm as a surrogate marker of *KRAS^G12D^* activation. In the control group (w/o *ela3I-CRE*), GFP signal was not detected. Asterisk (*) indicates the auto fluorescence of the gut. Scale bar: 25 µm.

To control *KRAS^G12D^* expression in a tissue specific manner, we generated a conditional *KRAS^G12D^* transgene under the control of the zebrafish *ubb promoter* with a lox-stop-lox (LSL) cassette inserted between the promoter and the start codon of the fusion *GFP-KRAS^G12D^* open reading frame (*ubb-Lox-Nucleus-mCherry-Lox-GFP-KRAS^G12D^*) (herein *LSL-KRAS^G12D^*) (Mosimann et al. 2011). We then crossed a female from the *Tg (LSL-KRAS^G12D^)* line with a male *Tg (elastase3I-CRE)* (herein *ela3I-CRE*) fish to enable pancreas-specific expression (Hesselson et al. 2011) (Figure 1(A)). To check the expression of *KRAS^G12D^* in the pancreas, we microdissected the pancreas from 5 dpf double transgenic *Tg (ela3I-CRE: LSL-KRAS^G12D^)* larvae. Nuclear mCherry signal was detected throughout the pancreas, confirming that the ubb promoter drives transgene expression ubiquitously in the vast majority of cell types (Mosimann et al. 2011) (Figure 1(B)). Accordingly, GFP signal was detected in the membrane of the pancreas because a single amino acid change at codon 12 of the human KRAS protein, from glycine (G) to asparagine (D), leads to membrane localization (Figure 1(B)). In the control group (w/o *ela3I-CRE*), we crossed a female from *Tg (LSL-KRAS^G12D^)* line to a male *AB* fish. Pancreas was microdissected from 5 dpf larvae. The nuclear mCherry signal was detected throughout the pancreas whereas the GFP signal as a surrogate marker of *KRAS^G12D^* activation was not (Figure 1(B)).

### Early *KRAS^G12D^*-responsive pancreatic progenitors contribute to endocrine as well as exocrine cells

**Figure 2.**
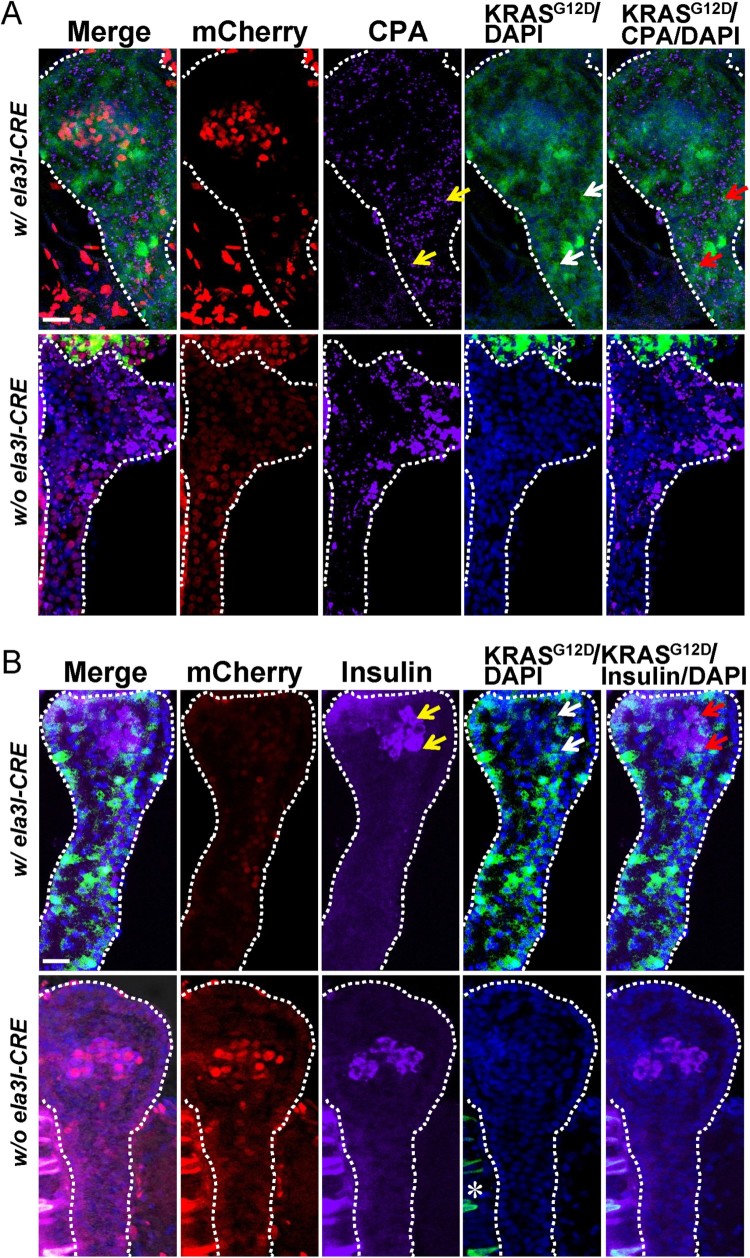
Early *KRAS^G12D^*-responsive pancreatic progenitors contribute to endocrine as well as exocrine cells. (A) The pancreas from 5 dpf double transgenic *Tg (ela3I-CRE; LSL-KRAS^G12D^)* larvae was stained with an exocrine specific marker, CPA. CPA staining was observed in the apical cytoplasm as well-developed apical secretory granules (yellow arrows). The GFP signal was detected in both the membrane and cytoplasm as a surrogate marker of *KRAS^G12D^* activation (white arrows). The merged confocal image showed that the pancreatic progenitor cells expressing oncogenic *KRAS^G12D^* expressed CPA in apical secretory granules of exocrine pancreas (red arrows). (B) The pancreas from 5 dpf double transgenic *Tg (ela3I-CRE; LSL-KRAS^G12D^)* larvae was stained with an endocrine specific marker, insulin. Insulin staining was shown in the cytoplasm of islet β-cells (yellow arrows). The merged confocal image showed that some of pancreatic progenitor cells expressing oncogenic *KRAS^G12D^* co-expressed the endocrine cell marker, insulin (red arrows). Asterisk (*) indicates the auto fluorescence of the gut. Scale bar: 25 µm.

A previous study showed that the *Tg (ela3I-CRE)* line specifically marked differentiated acinar cells in the normal condition (Hesselson et al. 2011). However, the *Tg (ela3I-CRE)* line promoted endocrine differentiation from the exocrine compartment under the low levels of *ptf1a* activity (Hesselson et al. 2011). To identify how early *KRAS^G12D^*-responsive pancreatic progenitors contribute to pancreatic cells, the pancreas from 5 dpf double transgenic *Tg (ela3I-CRE; LSL-KRAS^G12D^)* larvae were stained with an exocrine specific marker, CPA and an endocrine specific marker, insulin. CPA staining was observed in the apical cytoplasm as well-developed apical secretory granules (Figure 2(A), yellow arrows). As a surrogate marker of *KRAS^G12D^* activation, the GFP signal was detected in both the membrane and cytoplasm (Figure 2(A), white arrows). The merged confocal image showed that the pancreatic progenitor cells expressing oncogenic *KRAS^G12D^* expressed CPA in apical secretory granules of the exocrine pancreas (Figure 2(A), red arrows). In turn, insulin staining was observed in the cytoplasm of islet β-cells (Figure 2(B), yellow arrows). The merged confocal image showed that some of the pancreatic progenitor cells expressing oncogenic *KRAS^G12D^* co-expressed the endocrine cell marker, insulin (Figure 2(B), red arrows). Taken together, these data suggested that early *KRAS^G12D^*-responsive pancreatic progenitors contribute to endocrine as well as exocrine cells.

### Histological profiles of the abnormal pancreatic region

**Figure 3.**
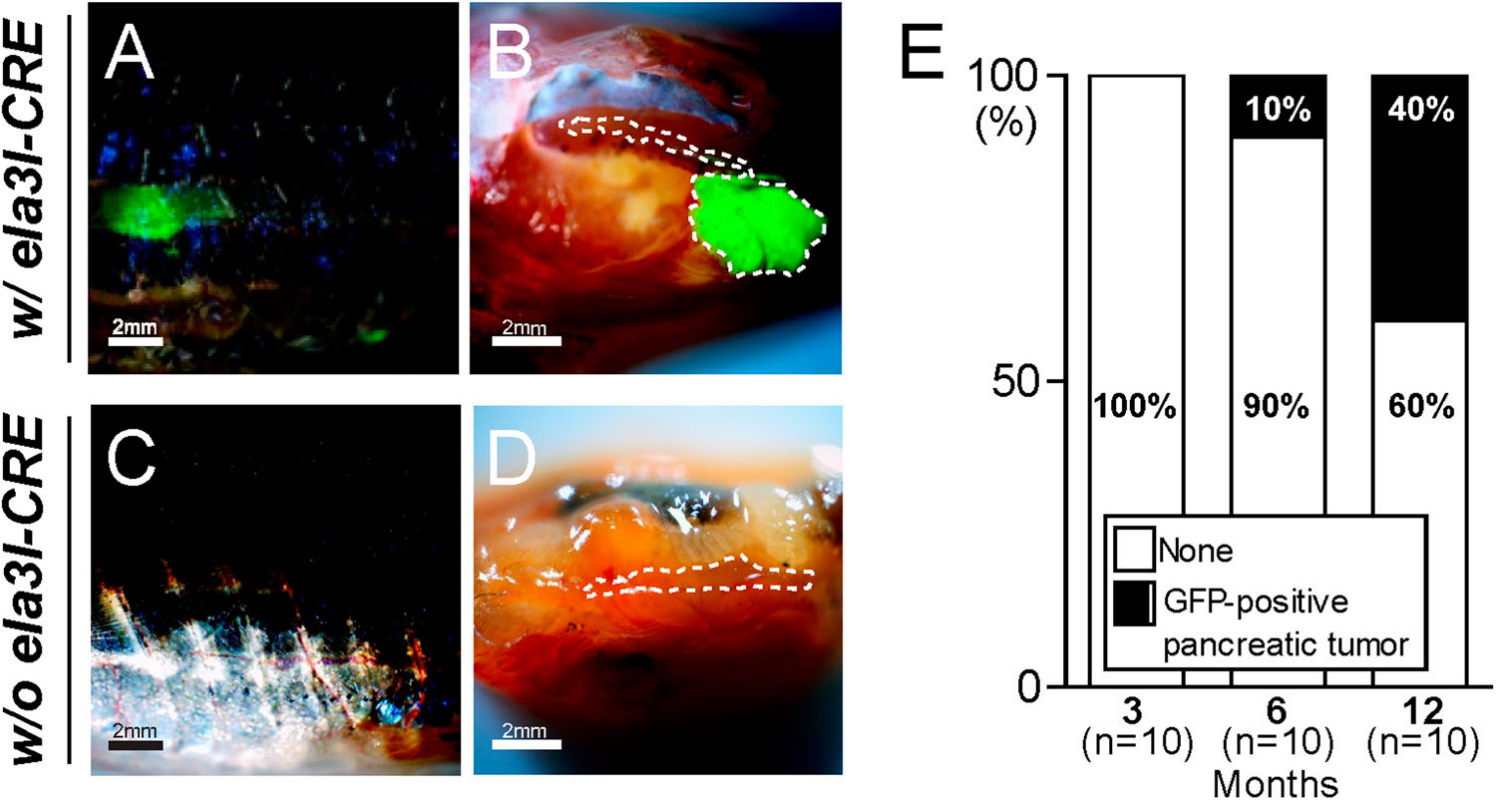
Identification of pancreatic tumors in *Tg (ela3I-CRE; LSL-KRAS^G12D^)* fish. (A and B) Transcutaneous fluorescence in the abdomen of *Tg (ela3I-CRE; LSL-KRAS^G12D^)* fish (A) and dissected abdominal viscera with a GFP-positive tumor in pancreas (B). (C and D) In the control group (w/o *ela3I-CRE*), no fluorescence was detected in the abdomen (C) and the dissected abdominal viscera (D). The dotted line indicated the pancreatic region. Scale bar: 2 mm. (E) Quantiﬁcation of GFP-positive pancreatic tumor frequency in *Tg (ela3I-CRE; LSL-KRAS^G12D^)* fish. A random subset of fish was anesthetized periodically at 3 (*n* = 10), 6 (*n* = 10), and 12 months (*n* = 10).

Tagging the oncogenic *KRAS^G12D^* with GFP allows tracing of pancreatic tumor formation by monitoring the visceral expression of GFP fluorescence in transgenic fish. A random subset of fish was anesthetized periodically and sacrificed at 3-, 6-, and 12-month time points. At 6 and 12- month time points, 1/10 fish (10%) and 4/10 fish (40%) developed transcutaneous abdominal GFP fluorescence (Figure 3(A)). Furthermore, GFP fluorescence in the pancreas was also observed from the dissected abdominal viscera (Figure 3(B)). This fluorescence was sufficiently strong to be distinguishable from autofluorescence in the intestinal tube or spleen under a fluorescence dissecting microscope (Figure 3(B)). However, the age-matched adult fish in the control group (w/o *ela3I-CRE*) did not show any transcutaneously-detectable GFP-positive lesions or GFP fluorescence in the pancreas (Figure 3(C and D)).

**Figure 4.**
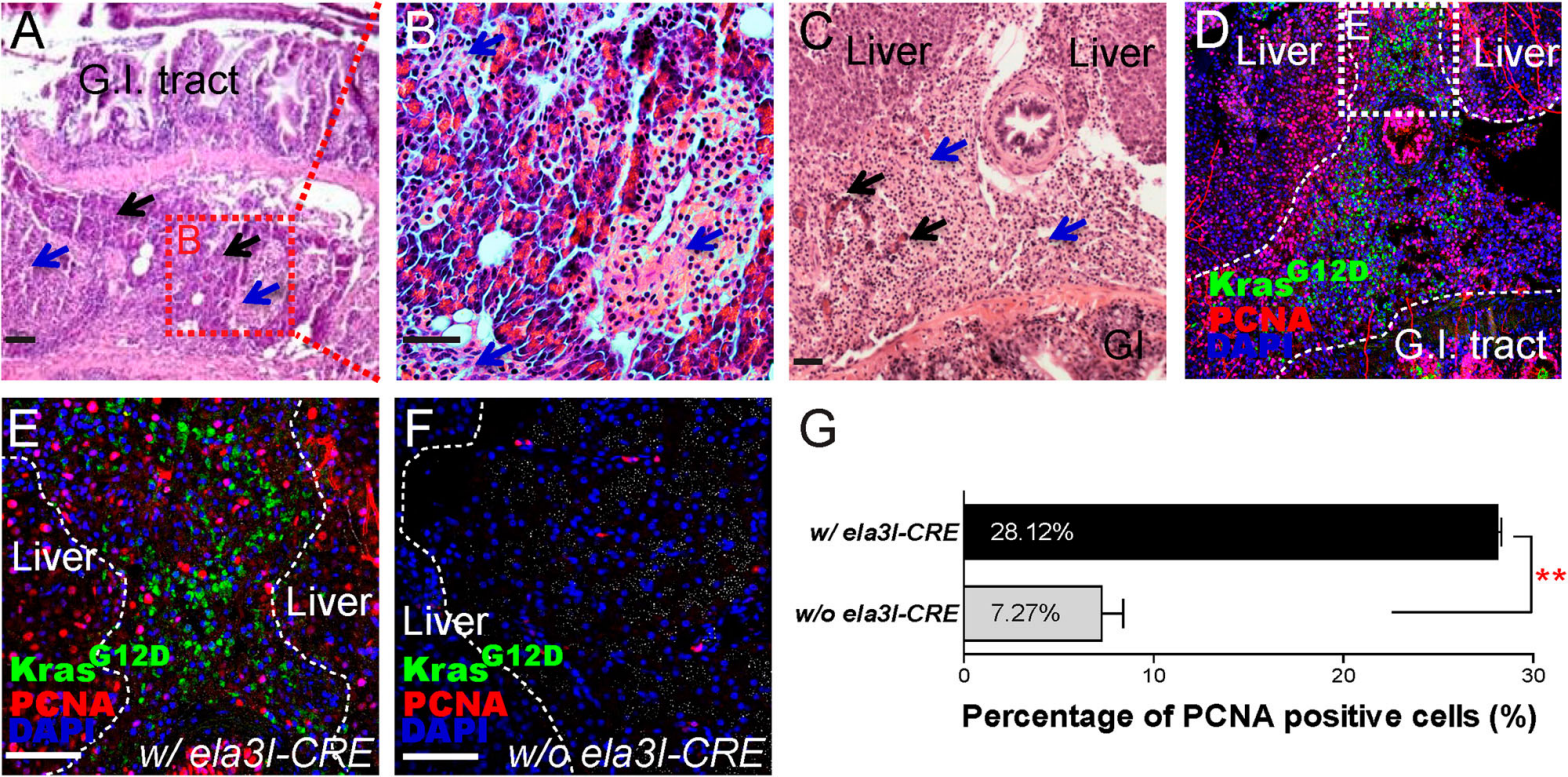
Histological profiles of the abnormal pancreatic region at 6 and 12 months of age. (A–C) Histological examination showed that acinar structure of pancreas (black arrows, zymogen granules in cytoplasm) is disrupted by expansion of populations of poorly differentiated round cells that resemble islet cells (blue arrows). Boxed areas indicate regions depicted at higher magnification in adjacent images. Scale bars: 50 μm. (D and E) GFP and PCNA staining in the abnormal pancreatic region. GFP and PCNA staining was observed in the poorly differentiated cells. Boxed areas indicate regions depicted at higher magnification in adjacent images. Scale bars: 50 μm. (F) In the control group (w/o *ela3I-CRE*), no GFP and infrequent PCNA staining was observed in the pancreas. Scale bars: 50 μm. (G) Comparison of PCNA positive cells. Highly frequent PCNA staining was observed in the poorly differentiated cells (w/ *ela3I-CRE*) (28.12%) compared to that in the control group (w/o *ela3I-CRE*) (7.27%) (**P < .01, Student’s *t*-test). Means ± S.D., *N* = 3. Gastrointestinal tract (G.I. tract).

Histological examination of the GFP-positive pancreas at 6 and 12 months of age showed that the acinar structure of pancreas (black arrows, zymogen granules in the cytoplasm) was disrupted through the expansion of poorly differentiated round cells (blue arrows) (Figure 4(A–C)). To identify the cell of origin, immunofluorescence was performed by using a GFP antibody. GFP signal was detected in the poorly differentiated cells, suggesting that oncogenic *KRAS^G12D^* induced the tumor phenotype (Figure 4(D and E)). To ascertain the effect of cellular proliferation from the expression of oncogenic *KRAS^G12D^* protein, PCNA staining was performed. PCNA signal was detected both in the poorly differentiated round cells (Figure 4(D and E)). However, the control group (w/o *ela3I-CRE*) showed no GFP expression and infrequent PCNA staining both in the pancreas (Figure 4(F)). When we compared the cellular proliferation between both groups, we found more proliferating cells in tissues with *ela3I-CRE* activation (28.12%) than in those without *ela3I-CRE* activation (7.27%) (Figure 4(G)).

### Immunohistochemical profile of the abnormal pancreatic region

**Figure 5.**
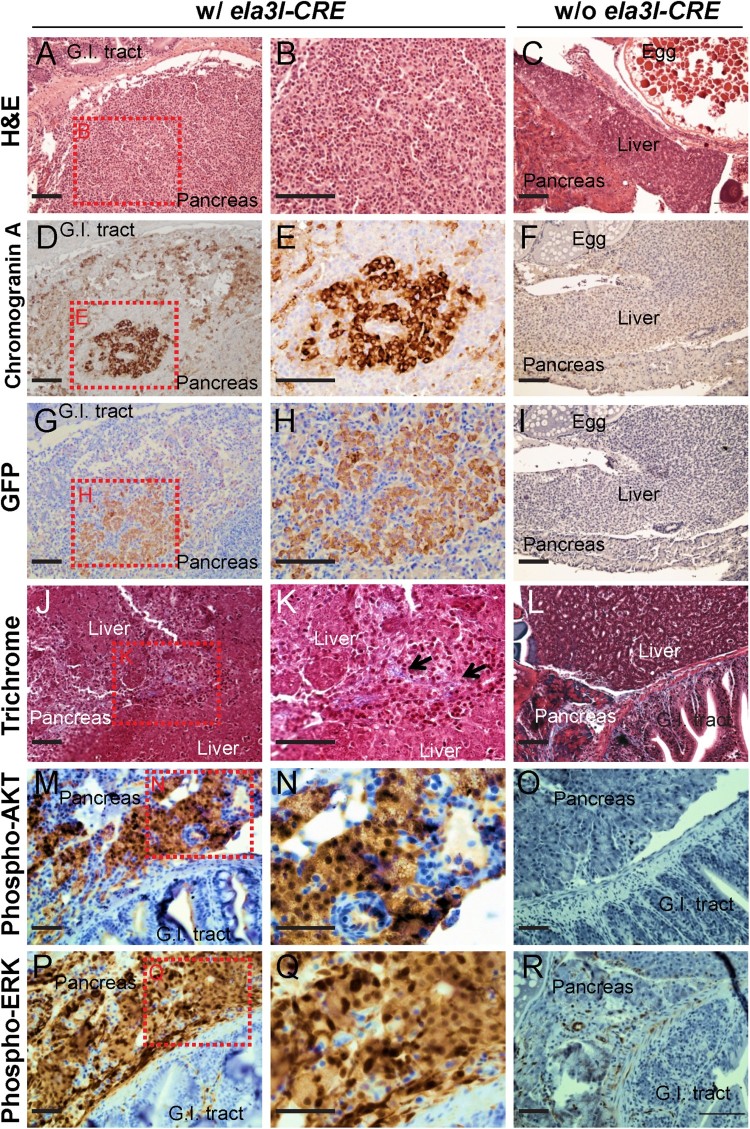
Immunohistochemical profiles of the abnormal pancreatic region at 6 and 12 months of age. (A–C) Histological profiles in *Tg (ela3I-CRE; LSL-KRAS^G12D^)* fish (A and B). Boxed areas indicate regions depicted at higher magnification in adjacent images. In the control group (w/o *ela3I-CRE*), normal histology was observed (C). Scale bars: 50 μm. (D–F) Enhanced chromogranin A staining was observed in the *KRAS^G12D^*-induced pancreatic tumor (D and E), whereas infrequent chromogranin A staining was observed in the endocrine region of the pancreas in the control group (w/o *ela3I-CRE*) (F). Scale bars: 50 μm. (G–I) The chromogranin A-positive tissues were also positive for GFP staining (G and H), whereas no GFP staining was observed in the control group (w/o *ela3I-CRE*). Scale bars: 50 μm. (J–L) Trichrome staining to identify the regions of fibrosis and sclerosis. The blue collagen fibers (black arrows) demonstrated that the invading carcinoma triggered fibrosis/sclerosis (J and K). In the control group (w/o *ela3I-CRE*), typical fibrotic changes were observed in the pancreas (L). Scale bars: 50 μm. (M–R) *KRAS^G12D^*-induced pancreatic tumors showed widespread labeling for both phospho-AKT (M and N) and phospho-ERK (P and Q), in contrast to infrequent AKT and ERK phosphorylation in controls (w/o *ela3I-CRE*) (O and R). Scale bar: 50 µm.

To identify the characteristics of the poorly differentiated round cells that resemble islet cells at 6 and 12 months of age (Figure 5(A and B)), immunohistochemistry was performed using the endocrine cell-specific marker, chromogranin A. Enhanced chromogranin A staining was observed in poorly differentiated round cells, indicating their endocrine cell-like characteristics (Figure 5(D and E)). In the control group (w/o *ela3I-CRE*), infrequent chromogranin A staining was observed in the pancreas where the endocrine cells were surrounded by the exocrine cells (Figure 5(F)). Next, GFP staining as a surrogate marker of *KRAS^G12D^* activation was performed. The chromogranin A-positive tissues were also positive for GFP, suggesting that the origin of the islet cell-like cells came from the cells expressing oncogenic *KRAS^G12D^* (Figure 5(G and H)). To further characterize whether pancreatic carcinoma triggered fibrosis/sclerosis, trichrome staining was performed. The blue collagen fibers (black arrows) demonstrated that the invading pancreatic carcinoma triggered fibrosis/sclerosis, which has been shown to be associated with pancreatic cancer progression (Dangi-Garimella et al. 2011; Olive et al. 2009) (Figure 5(J and K)). In the control group (w/o *ela3I-CRE*), typical fibrotic changes were observed in the pancreas between the liver and gut, where the exocrine pancreas surrounded the principal islets (Figure 5(L)).

To determine the status of downstream signaling pathways, which are known to be activated by oncogenic *KRAS^G12D^*, we assessed levels of phospho-AKT and phospho-ERK. In contrast to infrequent ERK and AKT phosphorylation in controls (Figure 5(O and R)), *KRAS^G12D^*-induced pancreatic tumors showed widespread labeling for both phospho-AKT and phospho-ERK (Figure 5(M, N, P, and Q)).

### Determination of the abnormal pancreatic region as pancreatic endocrine tumor

**Figure 6.**
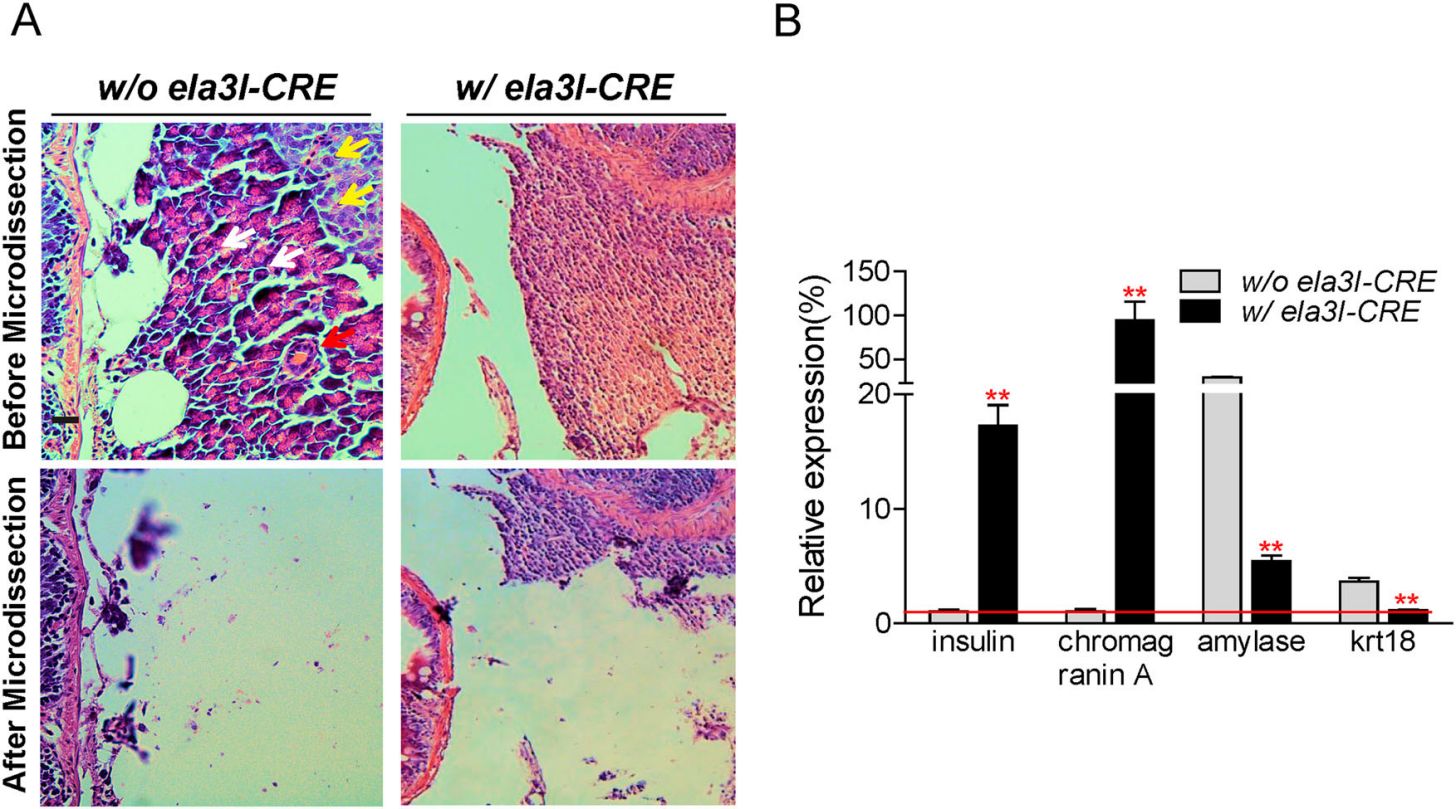
Molecular characterization of pancreatic tumors at 6 and 12 months of age. (A) To further classify the origin of pancreatic tumors, microdissection was performed. As a control, the pancreas region from the age-matched adult fish in the control group (w/o *ela3I-CRE*) was selected (exocrine cells: black arrows, endocrine cells: yellow arrows, ductal cells: red arrows). A representative example before/after microdissection was shown. Scale bar: 50 µm. (B) Quantitative PCR was performed against the mRNA for insulin, chromogranin A, amylase, and krt18 (**P < .01, Student’s *t*-test). Means ± S.D., *N* = 3.

The histological and immunohistochemical profiles indicated that *KRAS^G12D^*-induced pancreatic tumors were of endocrine origin. To further characterize these tumors, we microdissected the abnormal region from the paraffin-embedded sections at 6 and 12 months of age, and examined the gene expression level of exocrine, endocrine, and ductal markers. As a control, the pancreas region from the age-matched adult fish in the control group (w/o *ela3I-CRE*) was selected. The normal pancreas was composed of exocrine cell (black arrows), endocrine cell (blue arrows), and ductal cells (red arrows) (Figure 6(A)). A representative example before/after microdissection is shown in Figure 6(A). After isolating the RNA, quantitative PCR was performed against endocrine (insulin and chromogranin A), exocrine (amylase), and ductal (krt18) markers. The endocrine specific-markers, insulin and chromogranin A, were highly expressed compared to the control (Figure 6(B)). However, the exocrine (amylase) and the ductal (krt18) marker were highly expressed in the control but not in the *KRAS^G12D^*-induced pancreatic tumors (Figure 6(B)). Taken together, these results supported that *KRAS^G12D^*-induced pancreatic tumors could be classified as endocrine tumors.

## Discussion

In this study, we report the first *KRAS*-initiated pancreatic neoplasia model that closely recapitulates pancreatic endocrine tumors. This model shows the progression of pancreatic tumors with increased frequency along with advancing age. The histological and immunohistochemical profiles of these tumors bore a striking resemblance to those pancreatic endocrine tumors. However, a mouse model expressing mutant KRAS under the control of the elastase promoter led to the formation of invasive pancreatic ductal adenocarcinoma (Guerra et al. 2007). This discrepancy in cancer type may be partly explained by another mouse model, wherein the expression of a dominant-negative *Smad4* allele under the control of elastase:CreER^T2^ resulted in an age-dependent islet hypertrophy (Simeone et al. 2006). Furthermore, a transgenic mouse model expressing *c-myc* under the control of the *elastase* promoter exhibited only endocrine tumors with normal exocrine architecture (Lewis et al. 2003). These contradictory results raised an on-going debate regarding whether different pancreatic tumor types are derived from the transformation of distinct target cells or from common pancreatic progenitor cells. However, the lineage tracing results in the present study revealed that early *KRAS^G12D^*-responsive pancreatic progenitors contribute to endocrine as well as exocrine cells. To our knowledge, our study provides the first demonstration that pancreatic endocrine tumors are derived from the transformation of *KRAS^G12D^*-responsive pancreatic progenitor cells.

Pancreatic endocrine tumors comprise the second most common malignancy of the pancreas accounting for 1.3% of all cases of pancreatic cancer in incidence and 10% of cases in prevalence (Yao et al. 2008). Notably, pancreatic endocrine tumors pose signiﬁcant challenges in clinical treatment (Yao et al. 2011). Up to 50% of patients with pancreatic endocrine tumors have liver metastases at the time of diagnosis with median overall survival for such patients being only 24 months (Yao et al. 2011). However, no faithful animal model systems had been developed to evaluate different strategies for targeting pancreatic endocrine tumors. Conversely, our zebrafish model is well suited for testing preclinical strategies against pancreatic endocrine tumors. In particular, the optical clarity of embryos and larvae in our animal model allows real-time imaging of developing pathologies in pancreatic endocrine tumors. Specifically, a direct fusion between GFP and *KRAS^G12D^* makes it possible to monitor drug efficacy *in vivo* and to assess the severity of dedifferentiation or transdifferentiation. Thus, we propose that our zebrafish model may offer the possibility of an experimental and preclinical model system to evaluate different strategies for targeting pancreatic endocrine tumors and ultimately improve the outcome for patients with pancreatic endocrine tumors.

In summary, we successfully developed a novel system combining CRE/Lox technology to establish oncogenic *KRAS*-initiated pancreatic endocrine tumors. Our novel system demonstrated that *KRAS^G12D^*-responsive pancreatic progenitor cells could act as effective cells to induce pancreatic endocrine tumors. Thus, this zebrafish model provides an experimental and preclinical model system to investigate the basic biology of pancreatic endocrine tumors and identify potential therapeutic targets.
